# Direct image to subtype prediction for brain tumors using deep learning

**DOI:** 10.1093/noajnl/vdad139

**Published:** 2023-11-01

**Authors:** Katherine J Hewitt, Chiara M L Löffler, Hannah Sophie Muti, Anna Sophie Berghoff, Christian Eisenlöffel, Marko van Treeck, Zunamys I Carrero, Omar S M El Nahhas, Gregory P Veldhuizen, Sophie Weil, Oliver Lester Saldanha, Laura Bejan, Thomas O Millner, Sebastian Brandner, Sascha Brückmann, Jakob Nikolas Kather

**Affiliations:** Department of Medicine III, University Hospital RWTH Aachen, Aachen, North Rhine-Westphalia, Germany; Clinical Artificial Intelligence, Else Kroener Fresenius Center for Digital Health, Technical University Dresden, Dresden, Saxony, Germany; Department of Medicine III, University Hospital RWTH Aachen, Aachen, North Rhine-Westphalia, Germany; Clinical Artificial Intelligence, Else Kroener Fresenius Center for Digital Health, Technical University Dresden, Dresden, Saxony, Germany; Department of Internal Medicine I, University Hospital Carl Gustav Carus, Dresden, Saxony, Germany; Clinical Artificial Intelligence, Else Kroener Fresenius Center for Digital Health, Technical University Dresden, Dresden, Saxony, Germany; Department of Visceral, Thoracic and Vascular Surgery, University Hospital Carl Gustav Carus Dresden, Dresden, Saxony, Germany; Department of Medicine 1, Division of Oncology, Medical University of Vienna, Vienna, Vienna, Austria; Department of Pathology, St. Georg Teaching Hospital, University of Leipzig, Leipzig, Saxony, Germany; Clinical Artificial Intelligence, Else Kroener Fresenius Center for Digital Health, Technical University Dresden, Dresden, Saxony, Germany; Clinical Artificial Intelligence, Else Kroener Fresenius Center for Digital Health, Technical University Dresden, Dresden, Saxony, Germany; Clinical Artificial Intelligence, Else Kroener Fresenius Center for Digital Health, Technical University Dresden, Dresden, Saxony, Germany; Clinical Artificial Intelligence, Else Kroener Fresenius Center for Digital Health, Technical University Dresden, Dresden, Saxony, Germany; Neurology Clinic, Department of Neurology, National Center for Tumor Diseases (NCT), University Hospital Heidelberg, Heidelberg, Baden- Württemberg, Germany; Clinical Cooperation Unit Neuro-oncology, Department of Neurology, German Cancer Consortium (DKTK), German Cancer Research Center (DKFZ), Heidelberg, Baden- Württemberg, Germany; Department of Medicine III, University Hospital RWTH Aachen, Aachen, North Rhine-Westphalia, Germany; School of Medicine, Faculty of Medicine and Dentistry, University College London, London, Greater London, UK; Division of Neuropathology, Queen Square Institute of Neurology, University College London, London, Greater London, UK; Blizard Institute, Faculty of Medicine and Dentistry, Barts and The London School of Medicine and Dentistry, Queen Mary University of London, London, Greater London, UK; Division of Neuropathology, Queen Square Institute of Neurology, University College London, London, Greater London, UK; Institut für Pathologie, University Hospital Carl Gustav Carus, Dresden, Saxony, Germany; Department of Medicine III, University Hospital RWTH Aachen, Aachen, North Rhine-Westphalia, Germany; Clinical Artificial Intelligence, Else Kroener Fresenius Center for Digital Health, Technical University Dresden, Dresden, Saxony, Germany; Department of Internal Medicine I, University Hospital Carl Gustav Carus, Dresden, Saxony, Germany; Pathology & Data Analytics, Faculty of Medicine and Health, Leeds Institute of Medical Research at St James’s, University of Leeds, Leeds, West Yorkshire, UK; Department of Medical Oncology, National Center for Tumor Diseases (NCT), University Hospital Heidelberg, Heidelberg, Baden- Württemberg, Germany

**Keywords:** adult-type diffuse gliomas, deep learning, IDH, molecular signatures, subtype

## Abstract

**Background:**

Deep Learning (DL) can predict molecular alterations of solid tumors directly from routine histopathology slides. Since the 2021 update of the World Health Organization (WHO) diagnostic criteria, the classification of brain tumors integrates both histopathological and molecular information. We hypothesize that DL can predict molecular alterations as well as WHO subtyping of brain tumors from hematoxylin and eosin-stained histopathology slides.

**Methods:**

We used weakly supervised DL and applied it to three large cohorts of brain tumor samples, comprising *N* = 2845 patients.

**Results:**

We found that the key molecular alterations for subtyping, *IDH* and *ATRX*, as well as 1p19q codeletion, were predictable from histology with an area under the receiver operating characteristic curve (AUROC) of 0.95, 0.90, and 0.80 in the training cohort, respectively. These findings were upheld in external validation cohorts with AUROCs of 0.90, 0.79, and 0.87 for prediction of *IDH*, *ATRX*, and 1p19q codeletion, respectively.

**Conclusions:**

In the future, such DL-based implementations could ease diagnostic workflows, particularly for situations in which advanced molecular testing is not readily available.

**Key Points**

- Deep Learning can predict both molecular alteration status and subtype of diffuse adult-type gliomas directly from histopathology whole slide images.
- This is the first study to predict subtype and molecular status according to the 2021 WHO CNS 5th edition.

**Importance of the Study**

The 2021 update to the World Health Organization (WHO) classification system for central nervous system (CNS) tumors places further emphasis on molecular characterization in the diagnosis of adult-type diffuse gliomas. However, molecular assays are not necessarily available in all healthcare systems. Deep Learning (DL) offers an alternative approach for predicting molecular status directly from digitized histopathology slides. This study demonstrates that DL can accurately predict not only the subtype of glioma but also the status of individual molecular alterations. To our knowledge, this is the first study predicting clinically relevant markers according to the WHO fifth edition. This work provides further evidence supporting the use of digital workflows to support decision-making in clinical medicine.

Diffuse gliomas are the most frequent primary malignant brain tumors in adults and broad demographic shifts mean that their incidence is expected to increase.^1,2^ Due to the aggressive nature, infiltrative growth and central location, these tumors generally have a poor prognosis. Clinical outcome for these entities largely depends on subtype, the diagnosis of which has historically been based on histological assessment.^1,2^

In 2021, the World Health Organization (WHO) released the 5th edition of the diagnostic criteria for adult-type diffuse gliomas. These criteria are based on a combination of molecular alterations and histopathological assessment, which determines WHO CNS subtype and grade.^3^ In addition, methylation profiles can be used to define the glioma diagnosis.^2^

Based on their molecular profile, a significant proportion of adult-type gliomas can be subtyped by *IDH* (*IDH1* or *IDH2*) and *ATRX* mutation status, as well as 1p19q codeletion. Glioblastomas CNS WHO 4 are currently defined by the absence of *IDH* and *H3* mutations and occurrence of at least one of the following histopathological (microvascular proliferations, necrosis) or molecular (chromosome +7/−10 signature, *EGFR* amplification, *TERT* promoter mutation) alterations. *IDH*-mutant gliomas are subclassified into astrocytomas or oligodendrogliomas. The classification of *IDH*-mutant oligodendrogliomas CNS WHO 2/3 are defined by demonstration of a combined whole arm deletion of chromosomes 1p and 19q. Immunohistochemically retained nuclear *ATRX* expression as a surrogate of *ATRX*-wildtype status is a very strong correlate for 1p19q codeletion status in *IDH*-mutant gliomas.^2^*IDH*-mutant astrocytomas CNS WHO 2/3/4 are molecularly defined by retained 1p19q chromosomes and the presence of *ATRX*-mutation, or its immunohistochemical surrogate of nuclear *ATRX*-staining loss. The grading of *IDH*-mutant astrocytomas also relies on a combination of histomorphological, as well as molecular traits: the presence of *CDKN2A/B* homozygous deletion and/or necrosis and/or microvascular proliferation defines a CNS WHO grade 4 lesion in *IDH-mutant* astrocytomas.^2^

This evolution in the diagnostic approach represents a further shift away from the traditional histopathological process where pathologists typically assign subtypes based on specific tumor features, including morphology. The integration of genetic alterations into the diagnostic process reflects improved understanding of these tumors allowing for greater diagnostic precision. However, the necessity for molecular assessment and in some cases, methylation array profiling, also makes the process more expensive.^4^ These diagnostic requirements are further confounded by the fact that many advanced genetic tests are not available in all healthcare systems. For a fully integrated diagnosis, multiple different assays may be required to characterize different molecular features. This may further drive up costs associated with molecular testing, serving to widening the already pronounced inequities in precision medicine.^5^ Furthermore, the requirement for multiple tests could delay treatment by up to several weeks, with adverse sequelae for the patient.^6^

An increasing body of evidence indicates that Deep Learning (DL) techniques are able to predict the phenotypes linked to individual molecular alterations directly from routine hematoxylin and eosin (H&E) stained histopathology slides.^7^ This has the potential to accelerate diagnostic workflows and reduce the costs associated with molecular testing.^8^ These studies have shown that clinically relevant features, such as biomarkers and subtype, can be predicted directly from histopathology slides for many tumor types,^7,9,10^ including in brain tumors.^11,12^ DL is a machine learning (ML) method from the field of artificial intelligence (AI) and is a common and powerful way to extract quantitative information from image data.^13^ A typical DL workflow involves three main stages; preprocessing of the data, training and testing of an algorithm, and assessment of model performance through interpretation of statistical results.^13^

To assess the current status of DL research in diagnostic neuropathology, we performed a systematic literature review (Supplementary Figures S1 and S2). DL applications in brain tumors have mostly focused on classification tasks. The most common classification tasks consisted of molecular status prediction (*n* = 13) followed by grade (*n* = 10) and subtype prediction (*n* = 4). This demonstrates that brain tumors remain understudied in the field of computational pathology. This is particularly evident when compared to breast or colorectal cancer, which have been investigated in hundreds of computational pathology studies.^7^ The brain cancer-related studies identified via literature review were generally performed on small datasets,^11,14–16^ without external validation,^17–19^ or used networks that were not pretrained on histopathology images.^18,20–22^ This demonstrates a pressing need to address brain tumor research using larger, more heterogeneous cohorts, employing up-to-date methods. To our knowledge, there are no previously published studies attempting to predict subtype in adult-type diffuse gliomas on the basis of the WHO 2021 5th edition.

To this end, we collected and analyzed three independent datasets of digitized routine histological whole slide images (WSI) with the primary aims of predicting genetic alterations and differentiating brain tumor subtypes by using DL.

## Materials and Methods

### Ethics Statement

This study was performed in accordance with the Declaration of Helsinki. The research involved analysis of anonymized archival digital images of human tissue. Ethical approval was obtained at collaborating centers prior to collection and pseudonymization. Data were obtained from University College London as part of the UK Brain Archive Information Network (BRAIN UK), which is funded by the Medical Research Council and Brain Tumour Research. BRAIN UK reference number: 22-011—Artificial intelligence-based reconstruction of the WHO 2021 diagnostic algorithm for adult-type diffuse gliomas. The overall analysis was approved by the Ethics commission of the Medical Faculty of Technical University of Dresden (BO-EK-444102022). The STARD 2015 checklist can be found in Supplementary Table S2.

### Cohort Description

The first cohort was obtained from University College London (UCL) via Brain UK (REF:22/011). This cohort consisted of *n* = 1882 digitized H&E histopathology WSIs from *n* = 1877 patients with corresponding clinicopathological and molecular data. These are nonconsecutive cases collected from routine diagnostic work between 2011 and 2019. The dataset, curated from 40 sites across seven countries, encompasses both typical clinical cases and a small number of rare entities. All cases in which a diagnosis of an adult-type diffuse glioma was made were included in our study. These cases had an integrated molecular diagnosis according to WHO 2021, updated from a diagnosis according to WHO 2016, i.e., all cases were retrospectively assessed to comply with the CNS WHO 2021 classification.

The second cohort was derived from The Cancer Genome Atlas (TCGA, *n* = 864) network, www.cbioportal.org, for the tumor entities of low-grade glioma (LGG, *n* = 493) and glioblastoma (GBM, *n* = 371). The dataset is composed of cases contributed from 38 sites across seven countries. All cases in which a diagnosis of an adult-type diffuse glioma or a relevant genetic alteration was made, and were included in our study. Digitized histopathological WSIs with matching clinical–pathological and molecular data were obtained from www.cbioportal.org. Classification for this cohort was made according to WHO 2016. Molecular alteration data available for this cohort were used to update the subtype diagnosis to comply with WHO 2021, with assistance from an expert neuropathologist (SB). The third cohort was the Clinical Proteomic Tumor Analysis Consortium (CPTAC, *n* = 99) from https://www.cancerimagingarchive.net/collections/. All cases that were tested for *CDKN2A/B* homozygous deletion were included in our study. Matching clinicopathological and molecular data were downloaded from www.cbioportal.org. Data were obtained from these online sources as of March 13, 2022.

**Figure 1.**
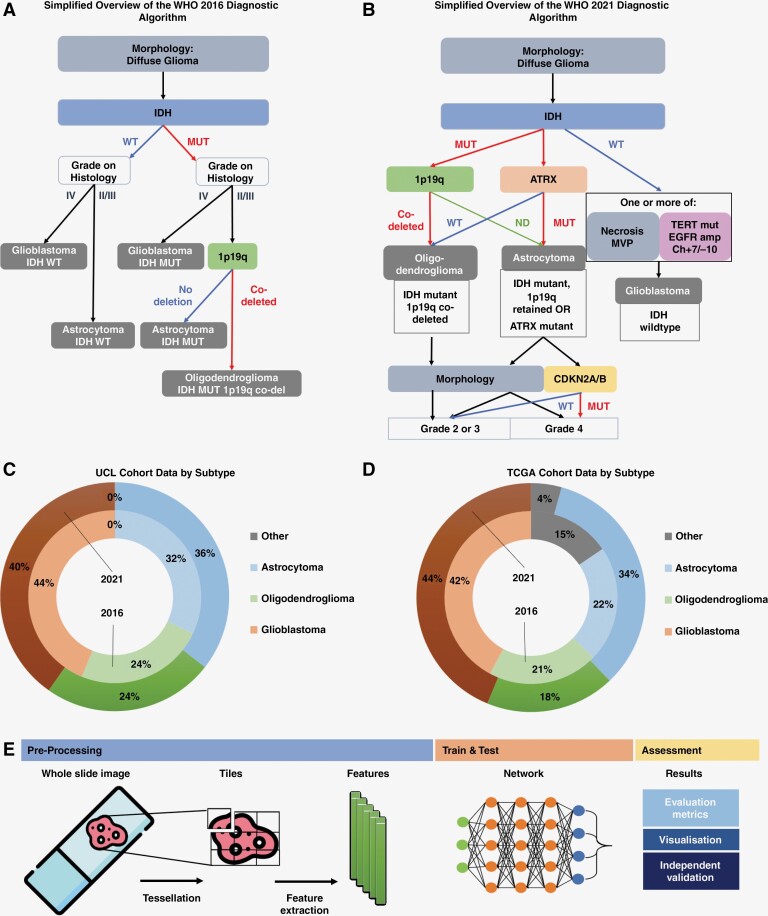
Overview of our experimental approach. The flow-chart Figure 1A and 1B outline the 2016 and 2021 WHO diagnostic algorithm for diffuse adult-type gliomas, for the targets that we included in our experiments. In the 2016 algorithm, gliomas were tested for *IDH* mutation status before the morphological features were assessed to determine grade. Both *IDHmut* and *IDHwt* tumors with high-grade (grade IV) features were designated glioblastoma. Tumors with lower grade (grade II or III) morphology were designated as astrocytoma, unless *1p19q* codeletion was present. Lower grade tumors with *IDHmut* and *1p19q* codeletion were designated oligodendrogliomas. In the 2021 system, molecular alteration status determines the subtype of glioma. Astrocytomas are *IDHmut* and *ATRXmut*. If *CDKN2A* or *CDKN2B* homozygous deletion are additionally present in an astrocytoma, this automatically upgrades the tumor to grade 4. Absence of *CDKN2A/B* homozygous deletion and absence of high-grade morphology indicates a grade 2 or 3 astrocytoma. Oligodendrogliomas are *IDHmut*, *1p19q* codeleted (complete loss of both arms), *TERTmut* and *ATRXwt*. Glioblastomas are *IDHwt* with either classic morphology on histology (microvascular proliferation and/or necrosis) or at least one of *TERTmut*, *EGFR* amplification, +7/−10 genotype. **Abbreviations:** MUT, mutated form; WT, wildtype form; ND, not deleted. The doughnut graphs in Figure 1C and 1D shows the data split by both 2016 and 2021 subtype diagnosis for the UCL and TCGA cohorts, respectively. Subtype diagnosis was available for both 2016 and 2021 criteria for the UCL dataset. In the TCGA dataset, the molecular alteration status for *IDH*, 1p19q and/or *ATRX* was used to formulate the 2021 subtype. Figure 1E provides an overview of our Deep Learning pipeline. The first step is preprocessing where digital WSIs are tessellated into tiles and features extracted for each tile. These features are then given to our network and used for training, testing, or deployment, depending on the type of experiment being run. Further details of our Deep Learning methods can be found in Supplementary Methods.

Information on the diagnostic process was sourced from WHO via their website https://tumourclassification.iarc.who.int/login. An overview of the neuropathological approach to diffuse adult-type gliomas can be found in Figure 1A and 1B. The diagnostic criteria informed which molecular data we collected for this study. Full details of the data collected and data preprocessing can be found in Supplementary Methods. An overview of the subtype data available in the UCL and TCGA cohorts can be found in Figure 1C and 1D. Further cohort data and consort charts can be found in Table 1 and Supplementary Figure S3, respectively.

**Table 1.** Provides information on the number of patients within each cohort for which genetic alteration data were collected. In the TCGA cohort, the low-grade glioma (LGG) and high-grade glioma (GBM) datasets were combined. As the subtype according to WHO CNS5 was not available in the TCGA dataset, molecular alteration data for *IDH*, 1p19q, and *ATRX* were used to formulate the WHO CNS5 diagnosis. This process was completed with guidance from a Neuropathologist. Consort charts for each cohort can be found in Supplementary Figure S2. The CPTAC cohort was only used in the *CDKN2A/B* external validation experiment and thus case numbers for the other mutations were not applicable.

		UCL, *n* = 1882			TCGA, *n* = 864			CPTAC, *n* = 99		
		Altered	Unaltered	N/d	Altered	Unaltered	N/d	Altered	Unaltered	N/d
Core diagnostic alterations	*IDH*	1116	755	11	410	318	136	N/a	N/a	N/a
	*ATRX*	584	887	411	207	521	136	N/a	N/a	N/a
	1p19q	316	683	883	162	542	160	N/a	N/a	N/a
Additional diagnostic alterations	*TERT*	524	277	1081	7	721	136	N/a	N/a	N/a
	*EGFR*	454	1115	313	207	650	7	N/a	N/a	N/a
	Ch + 7/-10	N/d	N/d	N/d	247	461	156	N/a	N/a	N/a
	*CDKN2A/B*	7	54	1821	266	592	6	56	40	3
WHO subtype 2016	Astrocytoma	600		7	180		280	N/a		
	Glioblastoma	822			231					
	Oligodendroglioma	453			173					
WHO subtype 2021	Astrocytoma	666		7	276^*^		148	N/a		
	Glioblastoma	756			279^*^					
	Oligodendroglioma	453			161^*^					

Ch +7/−10 indicates trisomy of chromosome 7 with monosomy of chromosome 10. For gene amplifications, altered represents amplification and unaltered no amplification. For chromosomal alterations, altered indicates deletion and/or gain and unaltered indicates normal ploidy.

^*^Subtype was formulated using molecular alteration data provided with the cohort.

Abbreviations: N/d, no data available; N/a, data not applicable.

### Deep Learning Methods

We have previously established^10,23^ and validated^24,25^ a DL pipeline to predict molecular alterations directly from histopathology images, including neuropathology.^26^ Here, we used an attention-based multiple-instance learning approach (attMIL).^26,27^ Preprocessing included normalization^28^ and tessellation of WSIs before features were extracted from each tile.^29^ The attMIL model makes predictions for a patient, based on a collection of tiles extracted from the patient’s slides. We call the aggregate of a patient’s features a *bag*, with the features itself being the bag’s *instances*. Because it is probable that not all of the instances have the same amount of information on the patient-level label, our model considers the entire bag at once. This enables it to consider information which may only be present in some of the instances while ignoring instances which contain little to no valuable information.^30^Figure 1E gives an overview of our approach. Full details of these methods can be found in Supplementary Methods. Our preprocessing and attMIL scripts are freely available on github (https://github.com/KatherLab/marugoto).

### Statistical Analysis

Model performance was assessed with the Area Under the Receiver Operating Curve (AUROC), which is the primary statistical endpoint in most studies on AI-based medical image analysis.^7^ A bootstrap 95% confidence interval (CI) is also given to provide a measure on AUROC accuracy. Furthermore, we provide additional statistics including sensitivity, specificity, accuracy and precision.

### Data and Code Availability

Access to the UCL data can be requested via the Brain UK study proposal platform at https://www.southampton.ac.uk/brainuk/index.page. Data from the TCGA and CPTAC cohorts can be accessed via the cBioPortal website (www.cbioportal.org or literature^31^).

### Experimental Design

Our aim was to use DL for subtype and molecular status prediction in adult-type diffuse gliomas according to the WHO 2021 5th edition. We investigated two approaches for subtype prediction:

1. Direct prediction: The tumor subtype is predicted from the WSI directly, according to both 2016 and 2021 WHO approaches. The 2016 results were used as the baseline from which to compare the 2021 results.
2. Sequential prediction: Each alteration in the diagnostic pathway is predicted separately. As a result of this output, the diagnostic pathway can be reproduced in a stepwise fashion, where the combination of alteration present and/or absent would then determine the subtype.

Both experimental approaches were run with internal cross-validation and external validation settings. The UCL cohort was chosen as the primary cohort for internal cross-validation and as a training set for external validation, due to having a larger number of patients and more balanced classes, with the exception of Ch +7/−10 and *CDKN2A/B*, as described in Supplementary Figure S3. Details of the cohorts that were used for each experiment are listed in Table 2.

**Table 2.** Results of internal and external validation experiments. This table provides results for all experiments and details of the cohorts used for each experiment. The molecular alteration experiments were run as individual experiments. The subtype experiments were run as one experiment, but results are listed for the prediction of each subtype individually. Experiments 1 to 13 were run in an internal validation setting. A five-fold internal cross-validation experiment was conducted, where the data are split randomly into five parts, with four parts being used for training the network and the remaining part used to test the model performance. This was repeated five times with the test data being rotated each time. Experiments 14 to 25 were external validation experiments, with training and deployment on the cohorts listed. For external validation, all data from one cohort were used to train the model and then deployed on an independent test cohort. We used UCL as a training cohort and TCGA and/or CPTAC as test sets, with the exception of *CDKN2A/B*, as we only had data within one cohort.

c	Exp. no.	Target	Train cohort	Deploy cohort	AUROC	95% CI
Internal validation experiments	1	2016 Astrocytoma	UCL	n/a	0.89	0.02
	2	2016 Glioblastoma	UCL	n/a	0.94	0.01
	3	2016 Oligodendroglioma	UCL	n/a	0.93	0.01
	4	2021 Astrocytoma	UCL	n/a	0.92	0.03
	5	2021 Glioblastoma	UCL	n/a	0.95	0.02
	6	2021 Oligodendroglioma	UCL	n/a	0.93	0.02
	7	*IDH*	UCL	n/a	0.95	0.02
	8	*ATRX*	UCL	n/a	0.91	0.03
	9	1p19q	UCL	n/a	0.80	0.03
	10	*TERT*	UCL	n/a	0.74	0.03
	11	*EGFR*	UCL	n/a	0.83	0.04
	12	Ch + 7/-10	TCGA	n/a	0.91	0.03
	13	*CDKN2A/B*	TCGA	n/a	0.82	0.02

	Exp. no.	Target	Train cohort	Deploy cohort	AUROC	95% CI
External validation experiments	14	2016 Astrocytoma	UCL	TCGA	0.86	0.03
	15	2016 Glioblastoma	UCL	TCGA	0.91	0.03
	16	2016 Oligodendroglioma	UCL	TCGA	0.86	0.04
	17	2021 Astrocytoma	UCL	TCGA	0.84	0.03
	18	2021 Glioblastoma	UCL	TCGA	0.90	0.02
	19	2021 Oligodendroglioma	UCL	TCGA	0.91	0.03
	20	*IDH*	UCL	TCGA	0.90	0.02
	21	*ATRX*	UCL	TCGA	0.79	0.04
	22	1p19q	UCL	TCGA	0.87	0.03
	23	*TERT*	UCL	TCGA	0.60	0.27
	24	*EGFR*	UCL	TCGA	0.85	0.03
	25	*CDKN2A/B*	TCGA	UCL + CPTAC	0.73	0.07

Experiments where the target is a 2016 subtype refers to gliomas that were diagnosed according to the 2016 WHO diagnostic guidelines. Likewise, experiments, where the target is a 2021 subtype, refer to gliomas that were diagnosed according to the 2021 WHO diagnostic guidelines. Further details on the diagnostic approaches can be found in Figure 1A and 1B.

Abbreviations: Exp. No., experiment number; AUROC, area under the receiver operating characteristic curve. AUROC is the rate at which a model can correctly predict the target. 95% CI, 95% confidence interval. The 95% CI is a range of the AUROC ± amount indicated by 95% CI. We can be 95% certain that the true AUROC falls within this range. Target, the feature that the network was aiming to predict in that experiment.

## Results

### Direct Prediction: 2016 and 2021 WHO Subtypes

The 2016 WHO subtypes of brain tumors are based on molecular alteration status plus histomorphology. DL utilizes the characteristics of an image to inform classification decisions. Hence, a DL model should be able to predict these subtypes from images more readily than the other targets. Once established, these results can be used as a baseline from which to assess our further experiments.

**Figure 2.**
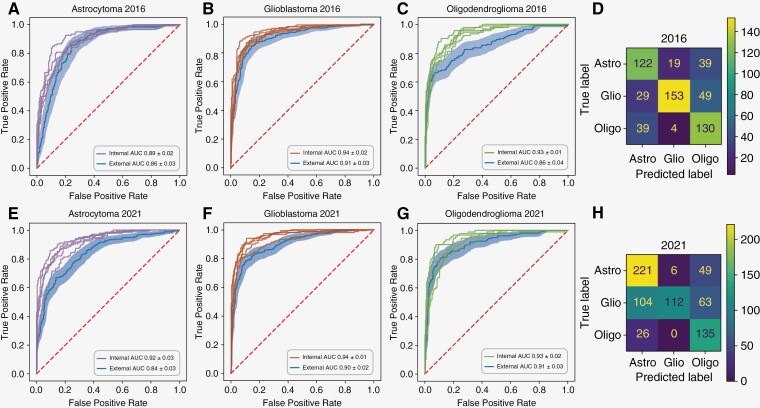
Results for subtype experiments. This figure shows Receiver Operating Characteristic (ROC) Curves and Confusion Matrices (CM) for both 2016 and 2021 subtype experiments. Subtype experiments were run as a single experiment. The AUROCs visualize results for each subtype individually, whereas the CMs and additional statistics in Figure 4 relate to overall model performance. CMs and additional statistics were calculated with a threshold of 0.5. In each ROC plot (A-C, E-G), the thin lines indicate ROC curves for internal validation experiments. Internal validation was performed as five-fold cross-validation. The line with shading indicates the external validation results; where the line is the external validation ROC curve and the shaded area around this line indicates the bootstrap CI. The AUC ± bootstrap CI is given in the bottom right of each plot. Please note, AUC refers to the area under the ROC curve and is thus the same as AUROC. D and H are heatmap confusion matrices for the 2016 and 2021 subtype experiments, respectively. The confusion matrices are constructed from the model prediction output for the external validation experiments, i.e., the class with the highest probability in external validation was selected as the predicted class.

In the cross-validation setting, our approach yielded AUROCs of 0.89 (CI ± 0.02), 0.94 (CI ± 0.01), and 0.93 (CI ± 0.01) for detection of astrocytoma, glioblastoma, and oligodendroglioma, respectively (experiments 1–3 in Table 2 and Figure 2). We also predicted the 2016 subtype in an external validation setting. These experiments yielded AUROCs of 0.86 (CI ± 0.03), 0.91 (CI ± 0.03), and 0.86 (CI ± 0.04) for astrocytoma, glioblastoma, and oligodendroglioma, respectively (experiments 14–16 in Table 2 and Figure 2).

We next investigated prediction of the 2021 subtype in an internal cross-validation, and our approach was able to yield AUROCs of 0.92 (CI ± 0.03), 0.95 (CI ± 0.02), and 0.93 (CI ± 0.02) for astrocytoma, glioblastoma, and oligodendroglioma, respectively (experiments 4–6 in Table 2 and Figure 2).

We then performed external validation, which gave AUROCs of 0.84 (CI ± 0.03), 0.90 (CI ± 0.02), and 0.91 (CI ± 0.03) for prediction of astrocytoma, glioblastoma, and oligodendroglioma, respectively (experiments 17–19 in Table 2 and Figure 2).

When comparing these results to our baseline 2016 results, the performances were very similar. The external validation AUROCs for astrocytoma and glioblastoma (2016 vs 2021) were within 0.02, and the internal validation AUROCs were within 0.03 and 0.01 for astrocytoma and glioblastoma, respectively (Figure 2A and 2B, 2E and 2G). For oligodendroglioma, the AUROCs remained congruous across the internal validation experiments (2016 vs 2021); however, on external validation, the AUROC for the 2021 experiment was 0.05 greater than in the 2016 experiment (Figure 2C and 2G).

### Sequential Prediction: Molecular Alterations

**Figure 3.**
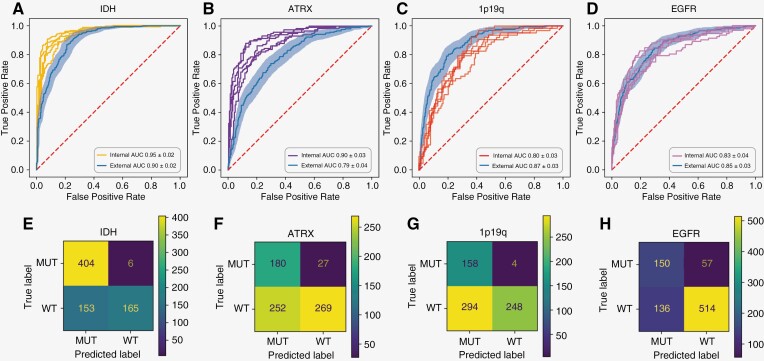
Results for molecular alteration experiments. (A)–(D) show Receiver Operating Characteristic (ROC) Curves for the molecular alteration experiments. In each ROC plot, the thin lines indicate ROC curves for internal validation experiments. Internal validation was performed as five-fold cross-validation. The line with shading indicates the external validation results; where the line is the external validation ROC curve and the shaded area around this line indicates the bootstrap CI. The AUC ± bootstrap CI is also given in the bottom right of each plot. Please note, AUC refers to the area under the ROC and is thus the same as AUROC. E–H are confusion matrices (CMs) for each molecular alteration experiment. The CMs are constructed from the model prediction output for the external validation experiments, i.e., the class with the highest probability in external validation was selected as the predicted class. CMs were calculated with a threshold of 0.5. **Abbreviations**: MUT, mutant; WT, wildtype; ALT, altered; UA, unaltered.

For our sequential prediction experiments, we began by predicting the core diagnostic molecular alterations (*IDH*, 1p19q, and *ATRX*) in a cross-validation setting. Prediction of *IDH* within this cohort was highly successful giving an AUROC of 0.95 (CI ± 0.02), with *ATRX* and 1p19q giving AUROCs of 0.91 (CI ± 0.03) and 0.80 (CI ± 0.03), respectively. For external validation of the core molecular alterations, results were similar to external validation for subtype prediction, with AUROCs of 0.90 (CI ± 0.02), 0.79 (CI ± 0.04), and 0.87 (CI ± 0.03) for *IDH*, *ATRX*, and 1p19q prediction, respectively (experiments 7–9 and 20–22 in Table 2 and Figure 3 A–C).

Furthermore, we performed subgroup analyses to ascertain how well the model was able to predict molecular alterations within a subgroup composed of each tumor subtype. However, unfortunately, our results did not find a strong link between any alteration and tumor subtype. ROC curves for these experiments can be found in Supplementary Figure S3.

We also aimed to investigate prediction of four additional molecular alterations; *TERT mutation*, *amplification of EGFR*, *CDKN2A/B homozygous deletion*, and chromosome trisomy 7 with monosomy 10 (ch +7/−10). For *EGFR amplification*, internal validation gave an AUROC 0.83 (CI ± 0.04) and external validation yielded an AUROCs of 0.85 (CI ± 0.03) (experiments 11 and 24 in Table 2 and Figure 3D). Unfortunately, we were unable to fully assess predictability of *TERT mutation*, *CDKN2A/B homozygous deletion*, and ch +7/−10 due to low prevalence of cases in our datasets. Preliminary results using the available data for these alterations are available in Supplementary Figure S4.

### Comparison of Approaches: Direct versus Sequential

**Figure 4.**
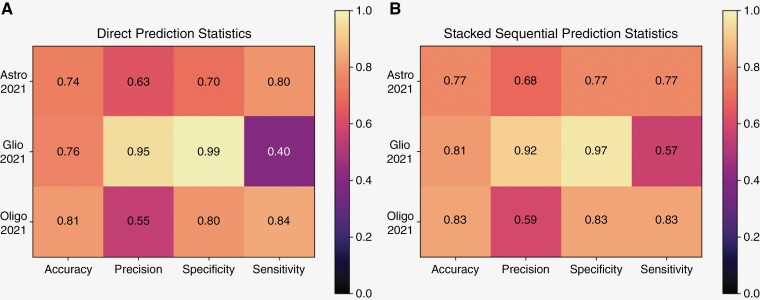
Statistical heatmaps. This is a heatmap of the further statistical analysis for our results. (A) shows results for the direct prediction of the 2021 subtypes. (B) shows results for the final prediction following the sequential approach. Here, the 2021 subtype was calculated by stacking the final predictions for the *IDH*, *ATRX*, and 1p19q experiments. Results for *IDH* prediction were considered first, followed by *ATRX* and 1p19q which were assessed together. Statistics were calculated with a threshold of 0.5.

In order to compare our two approaches, we stacked the external validation predictions for the three core alterations *IDH*, *ATRX*, and 1p19q to determine a final subtype prediction according to WHO CNS 2021. We then performed statistical analysis of these stacked predictions and compared them to statistical analysis of the external validation results for the 2021 direct prediction experiment (Figure 4). Overall, the results for the sequential prediction were superior to the direct prediction in all metrics (Figure 4), except for precision (0.92 in sequential, 0.95 in direct) and specificity (0.97 in sequential, 0.99 in direct) of glioblastoma prediction, and sensitivity for oligodendroglioma prediction (0.84 for direct and 0.83 for sequential). Excellent performance for specificity in both the direct and sequential approaches was noted.

### Interpretability: Plausible Features can be Linked to Predictions

To provide insight into the morphological features driving our network’s predictions, we produced heatmaps for a random sample of external validation WSIs for each target. These were then reviewed by an expert neuropathologist (SB, Supplementary Figure S7).

In the oligodendroglioma and 1p19q cases, so-called “fried egg” cells (densely packed cells with round nuclei and perinuclear clearing that resemble fried eggs) were consistently highlighted in our heatmaps, indicating that they are important for the prediction of these targets. This was the case for oligodendroglioma subtype prediction in both 2016 and 2021 experiments. Similarly, the *IDH*-wildtype and glioblastoma heatmaps (both 2016 and 2021 classifications) focused on areas of microvascular proliferation and pseudopalisading necrosis. Interestingly, *ATRX* mutation heatmaps were mostly “cold.” In some cases, gemistocytes appeared to be relevant to classification, but these cells were not present in all cases (Supplementary Figure S5).

## Discussion

The aim of this work was to recreate the diagnostic neuropathological workflow for diffuse adult-type gliomas with DL. Hence, we utilized a large dataset acquired through the Brain UK consortium—the UCL dataset—and demonstrated the efficacy of an attention-based DL pipeline on this task. We subsequently illustrated the general applicability of our pipeline by deploying on independent data using the publicly available TCGA and CPTAC datasets.

Through these methods, we were able to demonstrate that DL can infer both genetic alteration status and WHO subtype directly from WSI. Our results for subtype prediction and the three core diagnostic alterations were consistently above 0.79 in both internal and external validation. While an AUROC of 0.5 would indicate a random classifier, AUROCs of 0.7–0.8 indicate that a phenotype associated with the target is detectable to some degree by DL.^32^ Thus, we postulate that our model successfully identified and leveraged relevant phenotypes for these targets.

A variance in AUROC between internal- and external-validation experiments was notable for some targets. This is a common issue termed domain shift.^33^ Domain shift can occur due to multiple factors, particularly due to variations in tissue processing methods, staining and scanner properties between cohorts.^33^ While we employed multiple approaches to alleviate this problem, including training with a diverse cohort and normalizing tiles, model generalization remains an open problem in the field.^33^

Visual interpretability performed as part of this study highlighted morphologies that are recognized as being associated with specific subtypes as important to model decision-making. Fried egg cells and chicken-wire vasculature were found to correlate with prediction of 1p19q codeletion and oligodendroglioma subtype. Microvascular proliferation and necrosis correlated with *IDH-*wildtype and glioblastoma predictions, while gemistocytes and Rosenthal fibers correlated with astrocytoma and *IDHmut* predictions (Supplementary Figure S7). These features are used to make morphological diagnoses by neuropathologists.^2^ This indicates that our network used morphology in decision-making similarly to that of a neuropathologist and supports the correctness of our model.

Our study assessed two experimental approaches. Our direct approach predicted tumor subtype directly from WSI, whereas the sequential approach predicted mutational alteration statuses. Statistical analysis of these approaches indicated that overall, the sequential approach performed better. The sequential approach provides the added benefit of interpretability, as it allows pathologists to understand which alterations are present in a WSI, and thus support the final predicted subtype diagnosis. However, the excellent specificity of the direct approach for glioblastoma prediction should be noted. Glioblastoma is important to rule out in the clinical setting due to the poor prognosis.^34^

The limitations of our study primarily relate to our data. We were unable to evaluate all targets in the 2021 WHO CNS classification due to lack of data. Classes were particularly unbalanced for alterations such as *CDKN2A/B* in the UCL cohort and *TERT* in the TCGA and CPTAC cohorts, which can create biased predictions. Similarly, we were not able to externally validate ch +7/−10 as we only had data in the TCGA cohort. Furthermore, our study only included adult-type diffuse gliomas without considering other differential diagnoses, as would take place in clinical practice.

Further work would include acquiring more data from different sites. This would allow us to improve balance in our classes and provide more varied training data, including common differentials. This should help address the issue of domain shift and make our network more applicable to additional cohorts.

In conclusion, our study demonstrates that DL can predict the WHO CNS 2021 subtypes with high accuracy in a single and external cohort. Although a small number of studies have previously predicted WHO 2016 subtype and some molecular alterations, to our knowledge, no external validation experiments for 2016 subtype prediction have been previously performed. Furthermore, this is the first study to predict WHO 2021 subtype and the three core diagnostic alterations in one study.

## Supplementary Material

vdad139_suppl_Supplementary_Figures_S1-S7_Tables_S1-S2
