# Supplementary material for: Direct image to subtype prediction for brain tumors using deep learning
Source: Neurooncol Adv. 2023 Nov 1;5(1):vdad139. doi: 10.1093/noajnl/vdad139 (PMC10724115; doi:10.1093/noajnl/vdad139)
Supplement: vdad139_suppl_Supplementary_Figures_S1-S7_Tables_S1-S2 [file vdad139_suppl_supplementary_figures_s1-s7_tables_s1-s2.docx]

# **Supplementary Tables**

| **Classification** | | |
| --- | --- | --- |
| **Task** | **No. of Studies** | **References** |
| Molecular status prediction | 12 | 1, 2, 3, 4, 5, 6, 7, 8, 9, 10, 11, 12 |
| Grade prediction | 10 | 2, 13, 5, 14, 15, 16, 17, 18, 19, 20 |
| Subtype prediction | 4 | 21, 5, 4, 22 |
| Tissue type prediction | 2 | 23, 24 |
| Stage prediction | 1 | 25 |
| **Prognostication** | | |
| **Task** | **No. of Studies** | **References** |
| Survival prediction | 11 | 6, 8, 9, 10, 26, 27, 28, 29, 30, 31, 32 |
| Progression prediction | 2 | 8, 33 |
| Treatment response prediction | 2 | 9, 10 |

**Suppl. Table 1: Summary Table of Literature Review by Task.** This table summarizes the findings of our literature review. We identified 53 relevant papers which were categorized by task. Many papers performed more than one task and thus the sum of tasks performed exceeds the total number of included papers**.** The most common task-type performed by our included studies were classification tasks, specifically molecular status prediction.

| **Section & Topic** | **No** | **Item** | **Reported on page #** |
| --- | --- | --- | --- |
| **TITLE OR ABSTRACT** |  |  |  |
|  | **1** | Identification as a study of diagnostic accuracy using at least one measure of accuracy (such as sensitivity, specificity, predictive values, or AUC) | 1 |
| **ABSTRACT** |  |  |  |
|  | **2** | Structured summary of study design, methods, results, and conclusions  (for specific guidance, see STARD for Abstracts) | 2 |
| **INTRODUCTION** |  |  |  |
|  | **3** | Scientific and clinical background, including the intended use and clinical role of the index test | 2 |
|  | **4** | Study objectives and hypotheses | 3 |
| **METHODS** |  |  |  |
| *Study design* | **5** | Whether data collection was planned before the index test and reference standard were performed (prospective study) or after (retrospective study) | 4 |
| *Participants* | **6** | Eligibility criteria | 4 |
|  | **7** | On what basis potentially eligible participants were identified  (such as symptoms, results from previous tests, inclusion in registry) | 4 |
|  | **8** | Where and when potentially eligible participants were identified (setting, location and dates) | 4 |
|  | **9** | Whether participants formed a consecutive, random or convenience series | n/a |
| *Test methods* | **10a** | Index test, in sufficient detail to allow replication | 4 |
|  | **10b** | Reference standard, in sufficient detail to allow replication | n/a |
|  | **11** | Rationale for choosing the reference standard (if alternatives exist) | n/a |
|  | **12a** | Definition of and rationale for test positivity cut-offs or result categories  of the index test, distinguishing pre-specified from exploratory | n/a |
|  | **12b** | Definition of and rationale for test positivity cut-offs or result categories  of the reference standard, distinguishing pre-specified from exploratory | n/a |
|  | **13a** | Whether clinical information and reference standard results were available  to the performers/readers of the index test | n/a |
|  | **13b** | Whether clinical information and index test results were available to the assessors of the reference standard | n/a |
| *Analysis* | **14** | Methods for estimating or comparing measures of diagnostic accuracy | 5 |
|  | **15** | How indeterminate index test or reference standard results were handled | n/a |
|  | **16** | How missing data on the index test and reference standard were handled | n/a |
|  | **17** | Any analyses of variability in diagnostic accuracy, distinguishing pre-specified from exploratory | n/a |
|  | **18** | Intended sample size and how it was determined | n/a |
| **RESULTS** |  |  |  |
| *Participants* | **19** | Flow of participants, using a diagram | 20-22 |
|  | **20** | Baseline demographic and clinical characteristics of participants | n/a |
|  | **21a** | Distribution of severity of disease in those with the target condition | n/a |
|  | **21b** | Distribution of alternative diagnoses in those without the target condition | n/a |
|  | **22** | Time interval and any clinical interventions between index test and reference standard | n/a |
| *Test results* | **23** | Cross tabulation of the index test results (or their distribution) by the results of the reference standard | 6 |
|  | **24** | Estimates of diagnostic accuracy and their precision (such as 95% confidence intervals) | 6 |
|  | **25** | Any adverse events from performing the index test or the reference standard | n/a |
| **DISCUSSION** |  |  |  |
|  | **26** | Study limitations, including sources of potential bias, statistical uncertainty, and generalisability | 10 |
|  | **27** | Implications for practice, including the intended use and clinical role of the index test | 9, 18 |
| **OTHER INFORMATION** |  |  |  |
|  | **28** | Registration number and name of registry | n/a |
|  | **29** | Where the full study protocol can be accessed | n/a |
|  | **30** | Sources of funding and other support; role of funders | 10 |

**Suppl. Table 2: STARD 2015 Checklist**

#

# **Supplementary Figures**

**
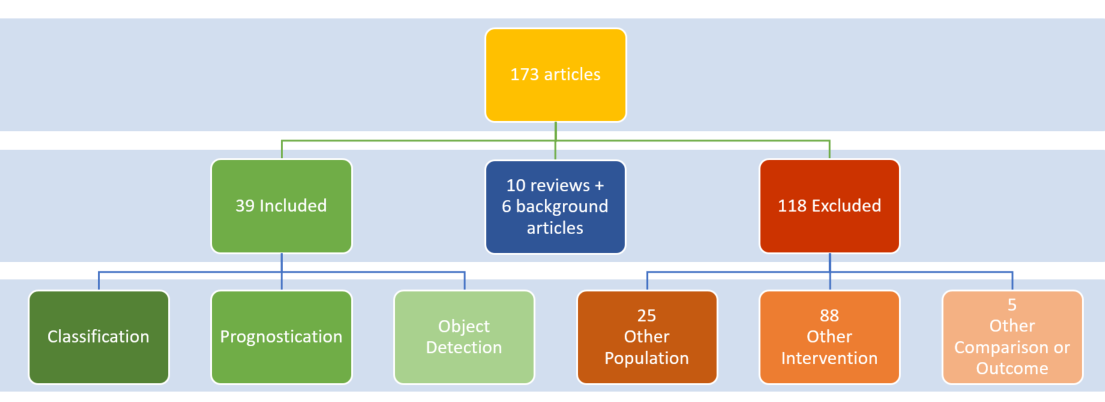
**

**Suppl. Figure 1: Flowchart for Literature Review.** Literature was identified using a PubMed search. Our search strategy can be found in **Suppl. Methods**. Included articles were reviewed using the PICO approach where:

Population - patients with primary adult-type diffuse glioma

Intervention - diagnosis of subtype and/or molecular status using histopathology H&E slides

Comparison - deep learning methods for determining subtype and/or molecular status

Outcome - accurate diagnosis according WHO 4th or 5th edition

**Suppl. Figure 3: Cohort data.** Table 1 provides information on the number of patients within each cohort for which genetic alteration data was collected. In the TCGA cohort, the low grade glioma (LGG) and high grade glioma (GBM) datasets were combined. Molecular alteration data was not always available for every patient in each cohort, thus the sum of altered and unaltered forms do not alway equal the total number of patients in the cohort. Consort charts for each cohort can be found in **Suppl. Fig. 2**. The CPTAC cohort was only used in the *CDKN2A/B* external validation experiment and thus case numbers for the other mutations were not applicable. Abbreviations: N/d = no data available. N/a = data not applicable. Ch+7/-10 indicates trisomy of chromosome 7 with monosomy of chromosome 10. For gene amplifications, altered represents amplification and unaltered no amplification. For chromosomal alterations, altered indicates deletion and/or gain and unaltered normal ploidy. * indicates that the WHO subtype was formulated. Formulated subtypes were calculated according to the relevant WHO guidance, using alteration and morphology data provided in the cohort, with supervision from a neuropathologist (SB).

**
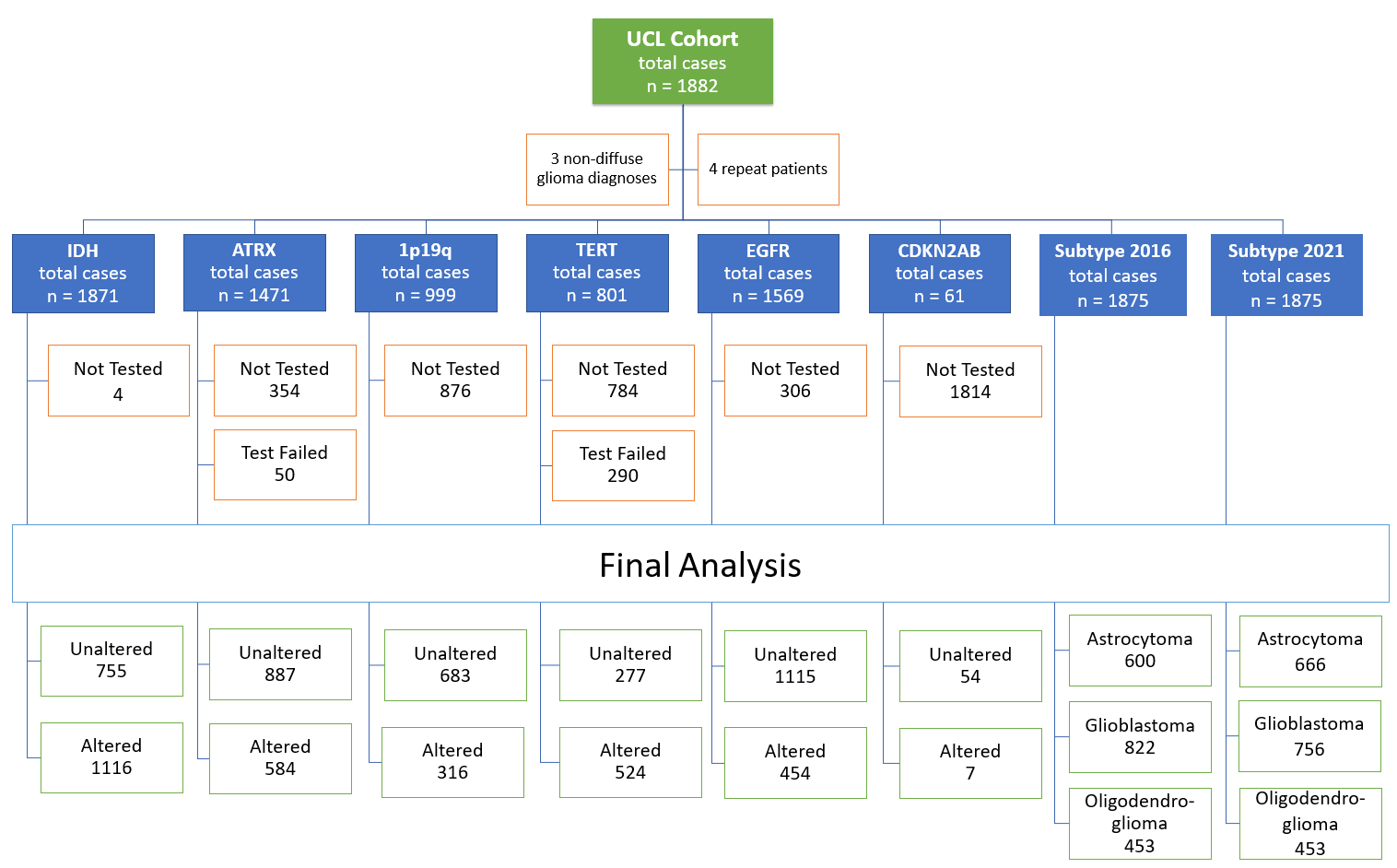
**

**Suppl. Figure 3a:** UCL consort chart. This flow diagram accounts for all cases within the University College London (UCL) cohort, received through our collaboration with BrainUK (REF:22/011).


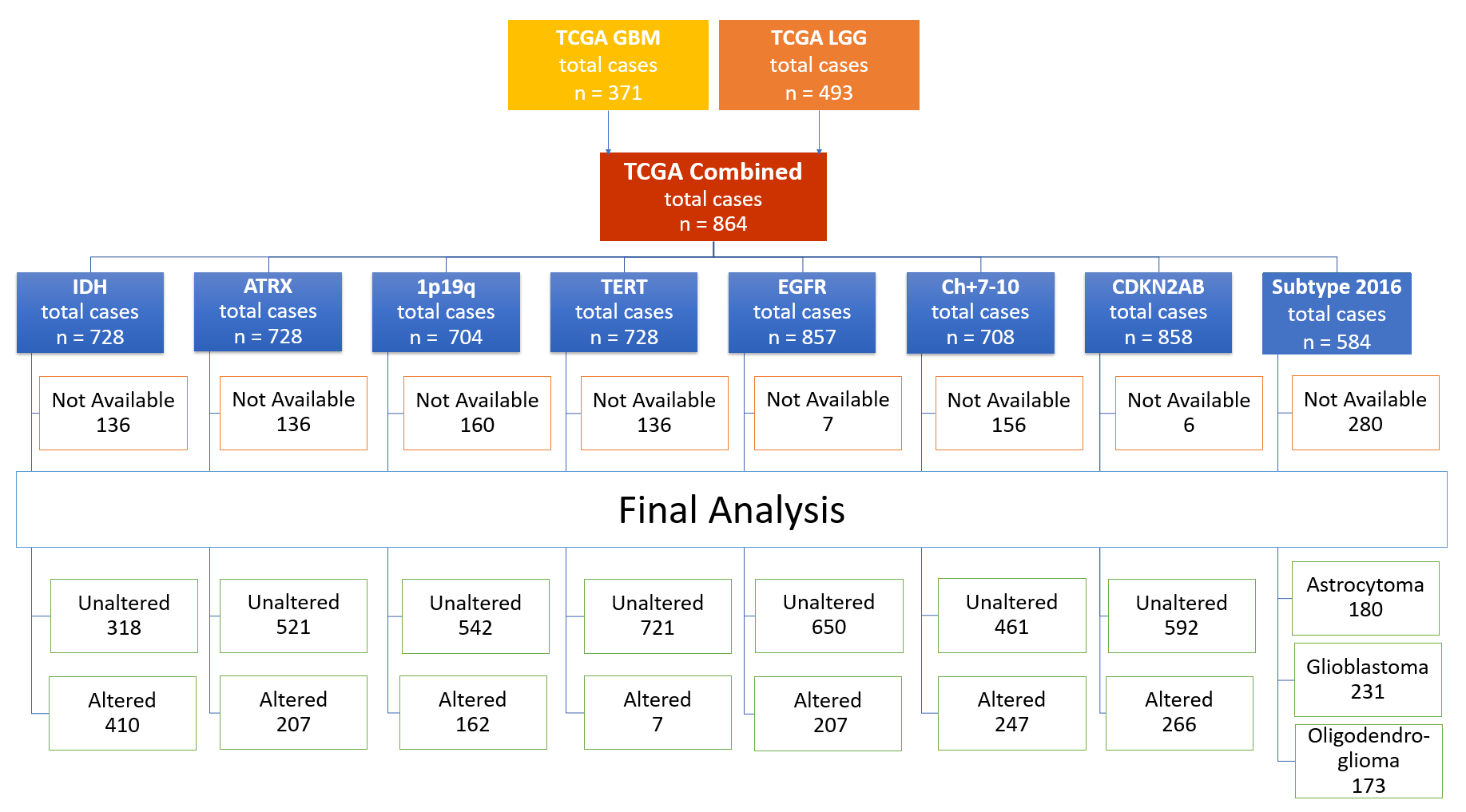


**Suppl. Figure 3b: TCGA Consort Charts.** This flow diagram accounts for all cases within the combined TCGA LGG and GBM cohorts.


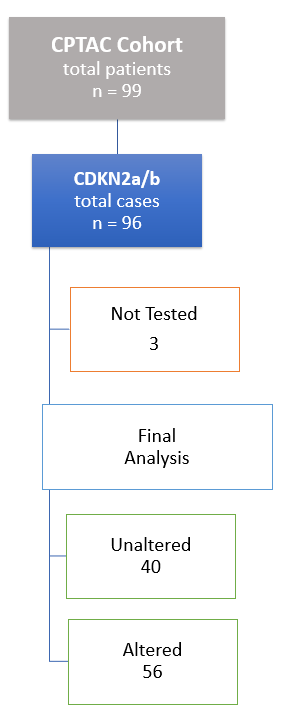


**Suppl. Figure 3c: CPTAC Consort Chart.** Only cases with *CDKN2A/B* homozygous deletion were used in our study. This flow diagram accounts for these cases within the CPTAC cohort.


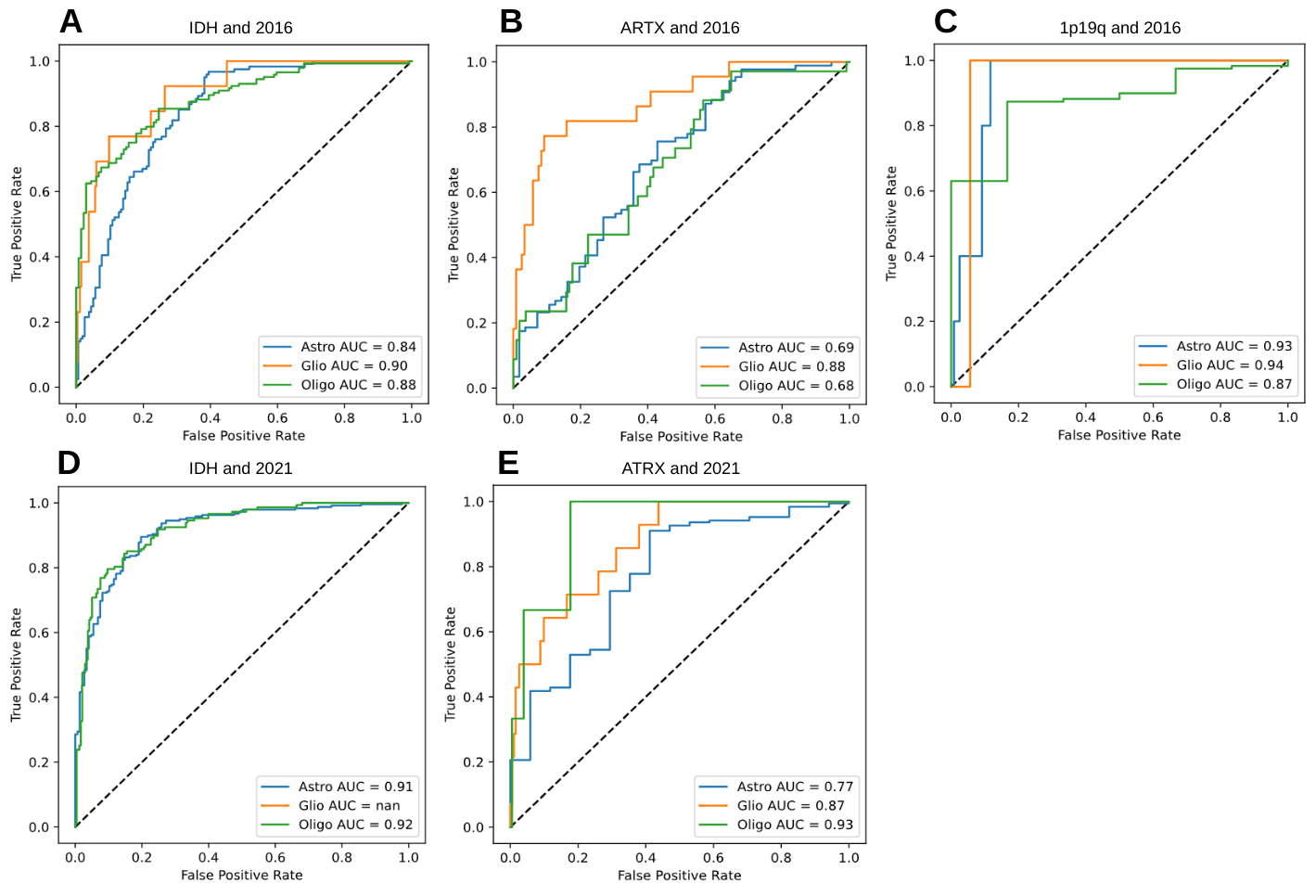


**Suppl. Figure 5:** **Subgroup analysis ROC curves.** We performed subgroup analysis to assess how well a specific molecular alteration could be predicted for each subtype. However, these results are inconclusive. ROCs A-C are results from predicting *IDH*, *ATRX* and 1p19q alteration status within the 2016 subtype. ROCs D and E are results from predicting *IDH* and *ATRX* in the 2021 subtype. Subgroup analysis for 1p19q was not possible in the 2021 classification as there were no glioblastoma or astrocytoma cases that harbored this alteration.

**
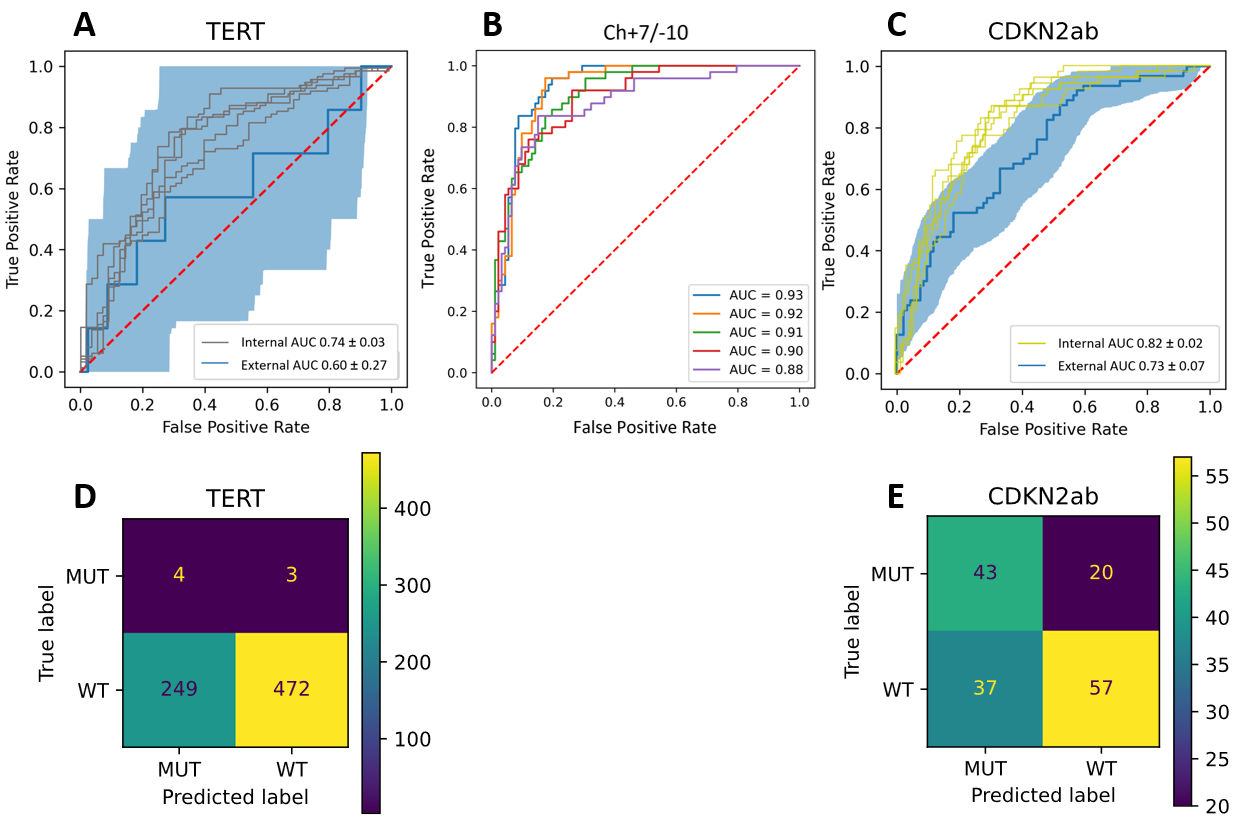
**

**Suppl. Figure 6:** Result for the additional molecular experiments. Figures A and C show the Receiver Operating Characteristic (ROC) Curves from the *TERT* and *CDKN2A/B* experiments. In these plots, the gray and yellow lines indicate ROC curves for internal validation experiments for *TERT* and *CDKN2ab* respectively. Internal validation was performed as five-fold cross-validation. The average Area Under the Curve (AUC) ± confidence interval (CI) for the internal validation experiments is given in the bottom right of each plot. The dark blue line in each plot indicates external validation results and the shaded area around this line indicates the CI. D and E are heatmap confusion matrices for the *TERT* and *CDKN2A/B* and experiments.

Figure B shows the ROC Curves for the chromosome +7/-10 internal validation experiment. Internal validation was performed as five-fold cross-validation. The average Area Under the Curve (AUC) ± confidence interval (CI) for these experiments are given in the bottom right of the plot. Please note, AUC refers to the area under the ROC curve, and is thus the same as AUROC.

For *TERT mutation* we used the UCL cohort for internal validation, which gave AUROCs 0.74 (CI±0.03). External validation was performed using TCGA, which yielded AUROCs of 0.60 (CI±0.27) for *TERT* (experiments 10 & 23 in Table 2 and Fig 3).

For *CDKN2A/B deletion*, the UCL dataset only included seven cases with deletion. Thus, we used the TCGA cohort for internal validation which gave an AUROC of 0.82 (CI±0.02). External validation for *CDKN2A/B deletion* was performed on the UCL dataset in combination with CPTAC and gave an AUROC of 0.73 (CI±0.07). These datasets were combined for this target only in an attempt to improve the balance of data classes.

For ch+7-10, we only had data in the TCGA cohort and therefore could only perform internal validation within the TCGA cohort. AUROC was 0.8 (CI±0.02) (experiment 12 in Table 2 and Suppl. Fig 4).

**I.**

**
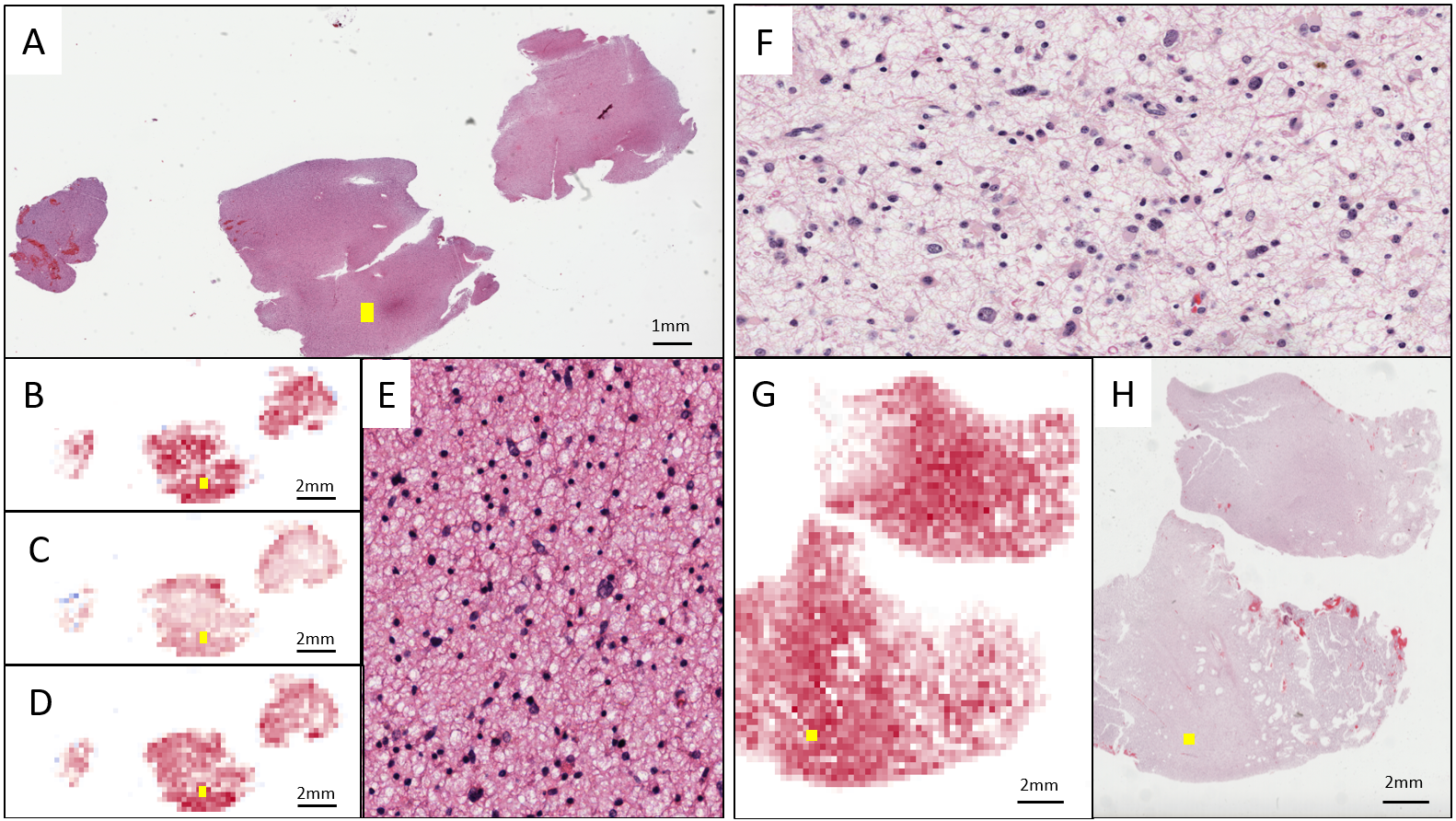
**

**II.**

**
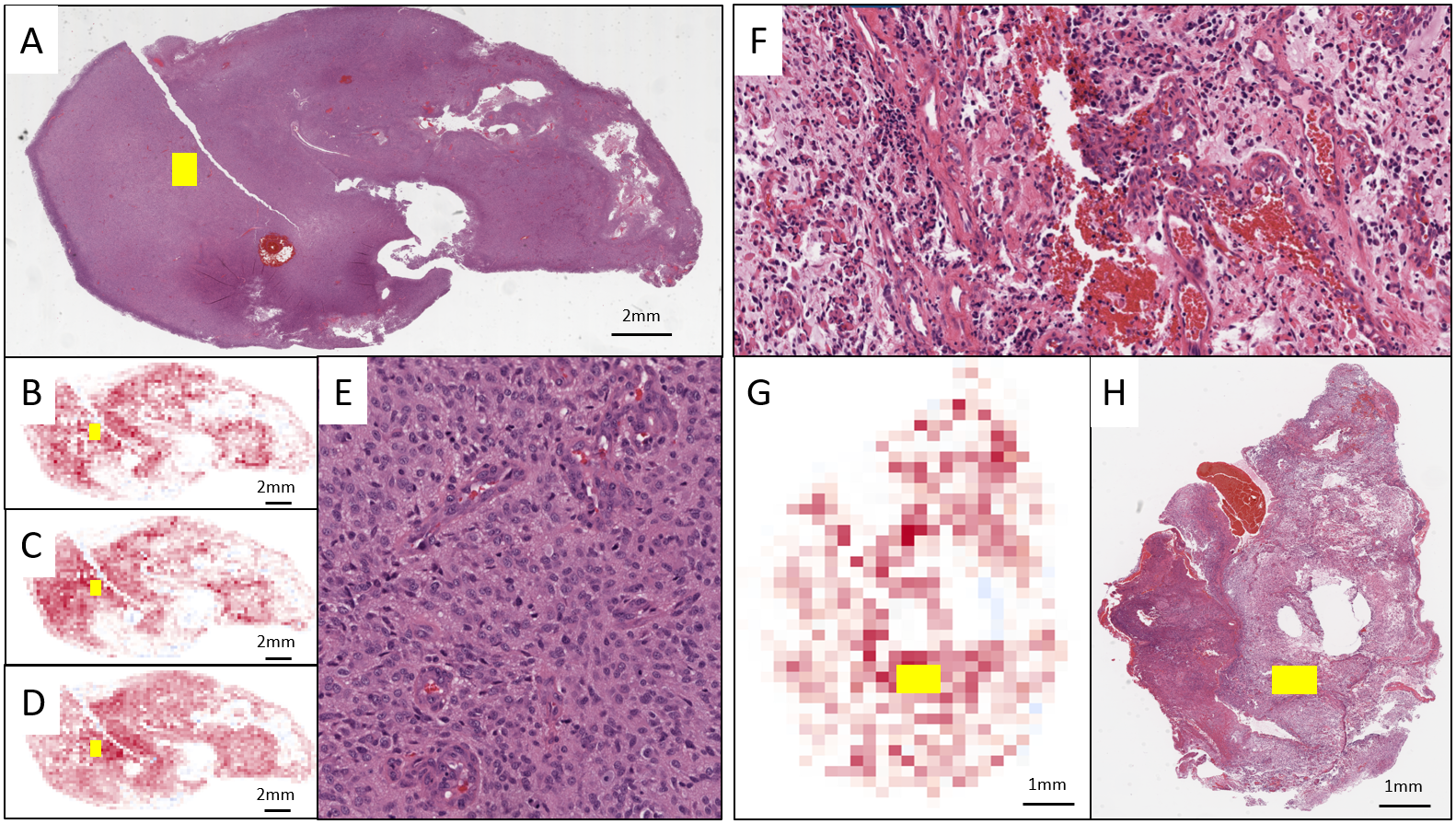
**

**III.**

**
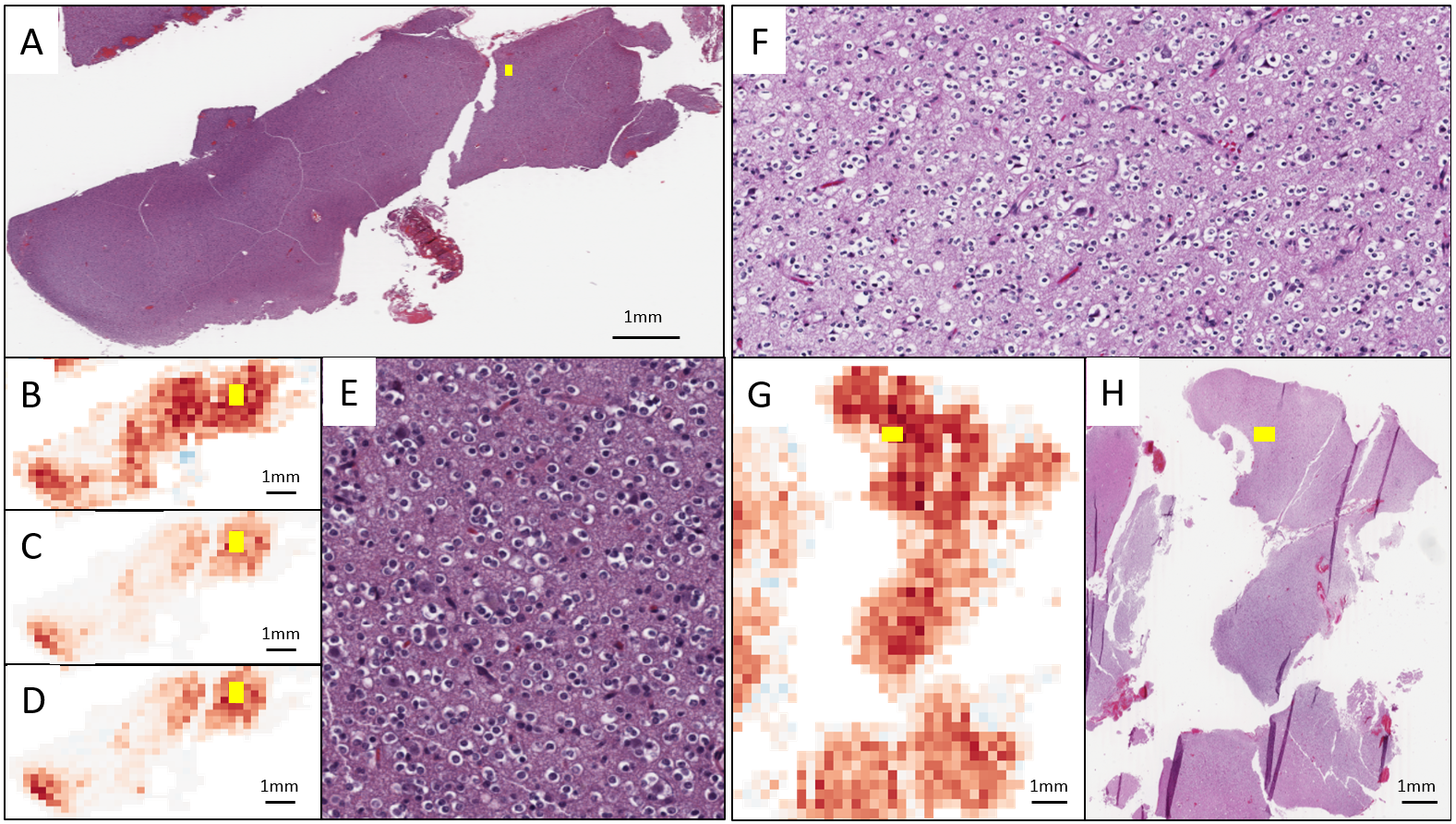
**

**Suppl. Figure 7: Heatmap Visualisation.**

Heatmaps provide topographical information based on the areas of the image the network used to make its prediction. Areas which appear red or ‘hot’ on the heatmap provided information that supported the positive class, whereas blue or ‘cold’ areas contained information relevant to the negative class. Areas without any color were not used to make any predictions.

1. Astrocytoma and *IDHmut* Visualization: A and H are low power images of two cases that were correctly predicted as astrocytoma on external validation in the 2016 and 2021 subtype experiments and as *IDHmut* in the IDH experiment. B, C and D are heatmaps from the IDH, 2016 and 2021 experiments respectively, for the WSI shown in A. G is a heatmap from the 2021 experiment for the WSI shown in H. E is a higher power view of the area indicated by the yellow boxes in A-D. F is a higher power view of the area indicated by the yellow box in G and H. Spongy neuropil, vacuolation (E) gemistocytes and Rosenthal fibers (F) were consistently noted in ‘hot’ areas on the astrocytoma and *IDHmut* heatmaps.
2. Glioblastoma and *IDHwt* Visualisation: A and H are low power images of two cases that were correctly predicted as glioblastoma on external validation in the 2016 and 2021 subtype experiments and as *IDHwt* in the IDH experiment. B, C and D are heatmaps from the IDH, 2016 and 2021 experiments respectively, for the WSI shown in A. G is a heatmap from the 2021 experiment for the WSI shown in H. E is a higher power view of the area indicated by the yellow boxes in A-D. F is a higher power view of the area indicated by the yellow box in G and H. Microvascular proliferation, nuclear pleomorphism (E), necrosis and anaplasia (F) were consistently noted in ‘hot’ areas on the glioblastoma heatmaps.
3. Oligodendroglioma and 1p19q Visualization: A and H are low power images of two cases that were correctly predicted as oligodendroglioma on external validation in the 2016 and 2021 subtype experiments and as 1p19q codeleted in the 1p19q experiment. B, C and D are heatmaps from the 1p19q, 2016 and 2021 experiments respectively, for the WSI shown in A. G is a heatmap from the 2021 experiment for the WSI shown in H. E is a higher power view of the area indicated by the yellow boxes in A-D. F is a higher power view of the area indicated by the yellow box in G and H. Cells with small, round nuclei and perinuclear clearing, so-called fried egg cells (E, F), and thin walled branching blood vessels (F), were consistently noted in ‘hot’ areas on the oligodendroglioma heatmaps.

#

# Supplementary Methods

## PubMed Search Strategy

We focused on identifying original research articles that applied a ML or DL method to histopathology images of primary adult-type diffuse gliomas, with the diagnosis made according to WHO 4th or 5th edition. We performed a search using PubMed, an extensive online database of research literature from biomedical academic journals. This search identified 173 articles, which were reviewed at title or abstract level to identify whether or not they were relevant to our work and ultimately, 53 articles were found to be pertinent. The search terms used to interrogate the database was as follows. PubMed search 05.09.22:

((((((((brain tumour[Title/Abstract]) OR (glioblastoma[Title/Abstract])) OR (oligodendroglioma[Title/Abstract])) OR (astrocytoma[Title/Abstract])) OR (glioma[Title/Abstract])) OR (glioma[MeSH Terms])) OR (brain tumor, primary[MeSH Terms])) AND ((((deep learning[Title/Abstract]) OR (machine learning[Title/Abstract])) OR (artificial intelligence[Title/Abstract])) OR (ai artificial intelligence[MeSH Terms]))) AND (((((pathology[Title/Abstract]) OR (histology[Title/Abstract])) OR (histopathology[Title/Abstract])) OR (neuropathology[Title/Abstract])) OR (histopathology[MeSH Terms]))

Limited to 2015 onwards = 149, reviewed at title/abstract = 53 relevant studies

The inclusion flow-chart is shown in Suppl Fig. 1.

## Data Preprocessing

Data from UCL, obtained through Brain UK, was received as a spreadsheet on 19.07.22. IDH mutation status was listed as mutated or wildtype (subtype was not specified), whereas TERT mutation status was specified as C228T, C250T or wildtype. TERT alterations C228T and C250T were grouped into mutated. EGFR amplification status was given as high, medium, low or no amplification. High, medium and low EGFR amplifications were grouped into altered. Copy Number Status (CpNS) data was provided for the chromosomes 1p36.12b, 1p13.3a, 19q13.42 and 19q13.2b. A CpNS of 1 indicated deletion, 2 indicated retained and 0 not tested. Cases where a CpNS of 1 was indicated for all four chromosomes constituted 1p19q co-deletion and these cases were grouped as altered. Data on the final diagnosis (morphological subtype plus IDH mutations status) according to the 2016 and 2021 WHO classifications were specified in separate columns. Free text from the neuropathological report was included with this dataset. This text listed ATRX mutation status (listed as mutated or wildtype) and CDKN2A/B as either deleted or not deleted. Free text data took precedence in the event of any discrepancies between other data.

Data for the TCGA and CPTAC cohorts was accessed via the cBioPortal website (www.cbioportal.org) on 19.03.2022. For these datasets, we downloaded copy number alteration (CNA) data for the genes EGFR, CDKN2A and CDKN2B, molecular alteration data for the genes TERT, IDH1, IDH2, ATRX for both cohorts. Copy Number Status (CpNS) data for the chromosomes 1, 7, 10 and 19 was only available for the TCGA cohort. Mutations were grouped into mutated (MUT) and wildtype (WT), and other alterations (such as 1p/19q, +7/-10, CDKN2A/B-del, EGFR-amp) were grouped into altered and unaltered. CDKN2A and CDKN2B alterations were called homozygous deletion with a value of -2 (loss than more than half of the baseline ploidy) and EGFR amplification was assigned with a value of +2 (gain of more than half of the baseline ploidy) https://docs.cbioportal.org/1.-general/faq#dna-mutations-copy-number- -fusions.

## Deep Learning Methods

Our pipeline uses attention-based multiple-instance learning (attMIL)17,18. Required pre-processing includes normalization, tessellation of WSIs and feature extraction. Normalization was performed using the Macenko19 approach, which uses statistical methods to reduce variation in image intensity and color, making the images within a dataset more consistent. Tessellation breaks the image down into smaller pieces called tiles. WSIs hold a large amount of information and it is computationally expensive to process the entire image in one step. Feature extraction converts each tile into a numerical representation and was performed using CTransPath20. CTransPath combines a convolutional neural network with a multi-scale Swin Transformer architecture and is pre-trained on unlabelled histopathology images using a self-supervised approach with contrastive learning20. The attMIL model makes predictions for a patient based on the features extracted from WSI tiles. We aggregate the patient’s tile features into a bag, where the features are a bag’s instances. Our model considers the entire bag at once as this enables it to consider information which may only be present in some of the instances while ignoring instances which contain little to no valuable information21. The network's structure is as follows: We project the tiles' features into smaller, 256-dimensional embeddings. These features are then fed to an attention mechanism consisting of two fully connected layers, which computes a scalar attention score for each tile. Each of the tiles' embeddings is then scaled with the softmax of the tile's attention score: the embeddings of tiles with low attention scores are diminished, while those with high attention scores are augmented. By summing up these scaled embeddings, we obtain a bag-level feature vector. Another fully connected layer then transforms this bag-level feature vector into a final classification.

## Visualization

In the misclassified cases, these often displayed features associated with a different tumor type. For example, in an astrocytoma case misclassified as oligodendroglioma, fried egg cells were present. Fried egg cells are characteristic for most oligodendrogliomas, however, this feature is not specific. Many tumor types other than oligodendrogliomas can contain fried egg morphology, and correspondingly, oligodendrogliomas without fried egg cells occasionally occur. Furthermore, in an astrocytoma case misclassified as glioblastoma, the heatmaps indicated that microvascular proliferation and necrosis played an important role in the classification. This morphology can be associated with both astrocytoma (grade 4) and glioblastoma subtypes (Suppl. Fig. 5G).

# Supplementary References

1. Liu, S. *et al.* Isocitrate dehydrogenase (IDH) status prediction in histopathology images of gliomas using deep learning. *Sci. Rep.* **10**, 7733 (2020).

2. Pei, L., Jones, K. A., Shboul, Z. A., Chen, J. Y. & Iftekharuddin, K. M. Deep Neural Network Analysis of Pathology Images With Integrated Molecular Data for Enhanced Glioma Classification and Grading. *Front. Oncol.* **11**, 668694 (2021).

3. Prokop, G. *et al.* Quantifying Heterogeneity in Tumors: Proposing a New Method Utilizing Convolutional Neuronal Networks. *Stud. Health Technol. Inform.* **289**, 397–400 (2022).

4. L, J. *et al.* Artificial intelligence neuropathologist for glioma classification using deep learning on hematoxylin and eosin stained slide images and molecular markers. *Neuro. Oncol.* **23**, 44–52 (2021).

5. S, I. *et al.* Classification of Diffuse Glioma Subtype from Clinical-Grade Pathological Images Using Deep Transfer Learning. *Sensors*  **21**, (2021).

6. Nalisnik, M. *et al.* Interactive phenotyping of large-scale histology imaging data with HistomicsML. *Sci. Rep.* **7**, 14588 (2017).

7. Ensenyat-Mendez, M., Íñiguez-Muñoz, S., Sesé, B. & Marzese, D. M. iGlioSub: an integrative transcriptomic and epigenomic classifier for glioblastoma molecular subtypes. *BioData Min.* **14**, 42 (2021).

8. Halani, S. H. *et al.* Multi-faceted computational assessment of risk and progression in oligodendroglioma implicates NOTCH and PI3K pathways. *NPJ Precis Oncol* **2**, 24 (2018).

9. Liu, X.-P. *et al.* Clinical Significance and Molecular Annotation of Cellular Morphometric Subtypes in Lower Grade Gliomas discovered by Machine Learning. *Neuro. Oncol.* (2022) doi:10.1093/neuonc/noac154.

10. Ji, H. *et al.* Novel Immune-Related Gene-Based Signature Characterizing an Inflamed Microenvironment Predicts Prognosis and Radiotherapy Efficacy in Glioblastoma. *Front. Genet.* **12**, 736187 (2021).

11. H, Z., A, M., Pl, C., H, V. & O, G. Whole slide images reflect DNA methylation patterns of human tumors. *NPJ genomic medicine* **5**, 11 (2020).

12. Cui, D., Liu, Y., Liu, G. & Liu, L. A Multiple-Instance Learning-Based Convolutional Neural Network Model to Detect the IDH1 Mutation in the Histopathology Images of Glioma Tissues. *J. Comput. Biol.* **27**, 1264–1272 (2020).

13. J, K., Y, B., Hy, L., J, R. & L, W. Automated brain histology classification using machine learning. *J. Clin. Neurosci.* **66**, 239–245 (2019).

14. Y, X. *et al.* Large scale tissue histopathology image classification, segmentation, and visualization via deep convolutional activation features. *BMC Bioinformatics* **18**, 281 (2017).

15. Truong, A. H., Sharmanska, V., Limbӓck-Stanic, C. & Grech-Sollars, M. Optimization of deep learning methods for visualization of tumor heterogeneity and brain tumor grading through digital pathology. *Neurooncol Adv* **2**, vdaa110 (2020).

16. S, R., T, N., Ma, I. & A, C. Glioma Grading via Analysis of Digital Pathology Images Using Machine Learning. *Cancers*  **12**, (2020).

17. X, W. *et al.* Machine Learning Models for Multiparametric Glioma Grading With Quantitative Result Interpretations. *Front. Neurosci.* **12**, 1046 (2018).

18. Komori, T. AI Neuropathologist: an innovative technology enabling a faultless pathological diagnosis? *Neuro-oncology* vol. 23 1–2 (2021).

19. Ertosun, M. G. & Rubin, D. L. Automated Grading of Gliomas using Deep Learning in Digital Pathology Images: A modular approach with ensemble of convolutional neural networks. *AMIA Annu. Symp. Proc.* **2015**, 1899–1908 (2015).

20. Nalisnik, M., Gutman, D. A., Kong, J. & Cooper, L. A. D. An interactive learning framework for scalable classification of pathology images. in *2015 IEEE International Conference on Big Data (Big Data)* 928–935 (2015).

21. Kurc, T. *et al.* Segmentation and Classification in Digital Pathology for Glioma Research: Challenges and Deep Learning Approaches. *Front. Neurosci.* **14**, 27 (2020).

22. Wang, X. *et al.* Combining Radiology and Pathology for Automatic Glioma Classification. *Front Bioeng Biotechnol* **10**, 841958 (2022).

23. K, F. *et al.* Visualizing histopathologic deep learning classification and anomaly detection using nonlinear feature space dimensionality reduction. *BMC Bioinformatics* **19**, 173 (2018).

24. G, B. *et al.* PathoFusion: An Open-Source AI Framework for Recognition of Pathomorphological Features and Mapping of Immunohistochemical Data. *Cancers*  **13**, (2021).

25. A, Y., H, K., Vbs, P., Bj, A. & H, T. Automatic disease stage classification of glioblastoma multiforme histopathological images using deep convolutional neural network. *Biomedical engineering letters* **8**, 321–327 (2018).

26. Hao, J., Kosaraju, S. C., Tsaku, N. Z., Song, D. H. & Kang, M. PAGE-Net: Interpretable and Integrative Deep Learning for Survival Analysis Using Histopathological Images and Genomic Data. *Pac. Symp. Biocomput.* **25**, 355–366 (2020).

27. Zadeh Shirazi, A. *et al.* A deep convolutional neural network for segmentation of whole-slide pathology images identifies novel tumour cell-perivascular niche interactions that are associated with poor survival in glioblastoma. *Br. J. Cancer* **125**, 337–350 (2021).

28. Rathore, S., Chaddad, A., Iftikhar, M. A., Bilello, M. & Abdulkadir, A. Combining MRI and Histologic Imaging Features for Predicting Overall Survival in Patients with Glioma. *Radiol Imaging Cancer* **3**, e200108 (2021).

29. Chunduru, P., Phillips, J. J. & Molinaro, A. M. Prognostic risk stratification of gliomas using deep learning in digital pathology images. *Neurooncol Adv* **4**, vdac111 (2022).

30. Rt, P. *et al.* Identification of Histological Correlates of Overall Survival in Lower Grade Gliomas Using a Bag-of-words Paradigm: A Preliminary Analysis Based on Hematoxylin & Eosin Stained Slides from the Lower Grade Glioma Cohort of The Cancer Genome Atlas. *J. Pathol. Inform.* **8**, 9 (2017).

31. P, M. *et al.* Predicting cancer outcomes from histology and genomics using convolutional networks. *Proc. Natl. Acad. Sci. U. S. A.* **115**, E2970–E2979 (2018).

32. Rj, C. *et al.* Pathomic Fusion: An Integrated Framework for Fusing Histopathology and Genomic Features for Cancer Diagnosis and Prognosis. *IEEE Trans. Med. Imaging* (2020).

33. Li, Z.-H. *et al.* Astrocytoma progression scoring system based on the WHO 2016 criteria. *Sci. Rep.* **9**, 96 (2019).
